# Development of Chitosan-Coated Atorvastatin-Loaded Liquid Crystalline Nanoparticles: Intersection of Drug Repurposing and Nanotechnology in Colorectal Cancer Management

**DOI:** 10.3390/pharmaceutics17060698

**Published:** 2025-05-27

**Authors:** Amina T. Mneimneh, Berthe Hayar, Sadaf Al Hadeethi, Nadine Darwiche, Mohammed M. Mehanna

**Affiliations:** 1 Pharmaceutical Nanotechnology Research Lab, Pharmaceutical Technology, Faculty of Pharmacy, Beirut Arab University, Beirut 11-5020, Lebanon; a.mneimneh@bau.edu.lb; 2 Department of Biochemistry and Molecular Genetics, American University of Beirut, Beirut 1107-2020, Lebanonnd03@aub.edu.lb (N.D.); 3 Department of Pharmaceutical Sciences, School of Pharmacy, Lebanese American University, Byblos 13-5053, Lebanon

**Keywords:** atorvastatin, Box–Behnken design, colorectal cancer, chitosan, cubosomes, nanoparticles

## Abstract

**Background:** Colorectal cancer (CRC) is the third most common cancer globally. Atorvastatin (ATR), a lipid-lowering drug, has shown promise as a repurposed therapeutic agent for CRC. However, its clinical application is limited by poor solubility and low oral bioavailability. This study aimed to optimize ATR-loaded chitosan-coated cubosomes using a Box–Behnken design and evaluate their potential in CRC treatment through physicochemical characterization and cell viability studies on HCT116 human CRC cells. **Methods:** Optimized cubosomes were characterized for particle size, zeta potential, polydispersity index (PDI), drug content, entrapment efficiency, compatibility using Fourier transform infrared spectroscopy, and in vitro release in various pH media. Cytotoxic effects were assessed using sulforhodamine B and trypan blue viability assays. **Results:** Uncoated cubosomes exhibited a particle size of 120.0 ± 1.66 nm, a zeta potential of −22.2 ± 1.05 mV, and a PDI of 0.136 ± 0.01. The chitosan-coated cubosomes displayed a size of 169.3 ± 4.14 nm, a zeta potential of 29.7 ± 0.814 mV, and a PDI of 0.245 ± 0.015. Entrapment efficiency and drug content were 92.1 ± 2.46% and 64.5 ± 1.58%, respectively. The ATR-loaded cubosomes demonstrated pH-dependent release, negligible at pH 1.2 and 4.5, but pronounced at pH 6.8 and 7.4, supporting colon-targeted delivery. Cell viability studies showed significant inhibition of HCT116 cells at ATR concentrations of 1 and 5 µM, with complete inhibition at higher doses. **Conclusions:** Chitosan-coated ATR-loaded cubosomes are promising for targeting ATR delivery to the colon. They offer enhanced anticancer activity by bypassing gastric degradation and systemic circulation, making an efficient approach to CRC treatment.

## 1. Introduction

Colorectal cancer (CRC) is a significant health problem that ranks third in terms of occurrence and second in terms of mortality worldwide [1,2]. According to the International Agency for Research on Cancer, the number of new cases is anticipated to increase with increasing life expectation. The American Cancer Society estimated 152,810 new CRC cases in both sexes, with 28,700 male and 24,310 female deaths [3].

There are several strategies to manage CRC, namely surgery, gene therapy, hormone therapy, radiotherapy, angiogenesis suppressing, and chemotherapy [4,5]. The choice of treatment is solely dependent on the stage of colon cancer. During the early stages, where cancer is still localized in the colon and has not grown further, surgery is the ultimate treatment. In contrast, surgery is often accompanied by chemotherapy in more advanced stages, as in stages II and III. However, for patients in stage IV, the surgical approach is impractical and does not provide the intended outcome because the cancer has metastasized, so chemotherapy is their only option [6].

Even though cancer management via chemotherapy is one of the most effective and persuasive approaches, uncontrollable metastasis accelerates tumor spread, and the prolonged treatment duration allows cancer cells to develop resistance toward chemotherapeutic agents, leading to poor prognosis [7,8]. A multicenter cohort study in Lebanese hospitals showed that targeted therapy accompanied by standard therapy is highly prevalent in Lebanon in metastatic disease, and the associated medical costs are substantial. This study proved targeted therapy’s clinical effectiveness and costs in patients with metastatic colorectal cancer [9]. In addition, designing and testing new anticancer agents is time-consuming, with an average of 13 years between cellular studies, animal assessments, and human trials, and a substantial cost of USD 2–3 billion [10].

The application of atypical medications as anticancer-repurposed therapeutics has gained an increasing interest in the oncology field. Using existing data on safety and toxicity, the ready availability of such medicines and the potential to lower drug costs support the drug repurposing approach [8]. The ReDo project (Repurposing Drugs in Oncology) is an ongoing international collaboration focused on the potential use of approved non-cancer medications as a foundation for new cancer therapeutics management approaches. This project has included 268 repurposed drugs in its database system, comprising summary results and an assessment of the clinical trial activity of these drugs. This database has been available as an online open-access resource [11]. Cannabinoids such as cannabidiol (CBD) and tetrahydrocannabinol (THC) have shown potential in cancer treatment. A CBD/THC mixture from Lebanese Cannabis sativa effectively reduced breast cancer cell growth and motility, inducing apoptosis through mitochondrial and caspase-dependent pathways [12]. In addition, low-dose β-2-himachalen-6-ol (HC) oil derived from the Lebanese wild carrot led to cell cycle arrest and apoptosis, along with significant antitumor activity in vivo, without causing major toxicity in mice [13]. These results emphasize the promise of cannabinoids and HC as safe, non-conventional therapeutic options for treating aggressive and resistant cancers.

Another example is statins. Statins are 3-hydroxy-3-methylglutaraldehyde coenzyme A (HMG-CoA) reductase inhibitors that efficiently block the conversion of HMG-CoA into meglutarate, thus suppressing the synthesis of cholesterol. Statins are traditionally used to treat hyperlipidemia and prevent cardiovascular diseases [14]. Elevated serum cholesterol levels have been positively linked with the risk of developing cancers, particularly colon, rectal, prostatic, and testicular cancer [15,16]. Statins are potential candidates for eliciting their anticancer effects through cholesterol depletion. Lipophilic statins such as simvastatin, atorvastatin (ATR), lovastatin, and pitavastatin have shown superiority in penetrating cancer cells. They are associated with a better prognosis in patients with CRC cancer, breast cancer, hepatocellular carcinoma, and prostate cancer compared to hydrophilic statins such as pravastatin and rosuvastatin [17]. Ongoing clinical trials are presently investigating the efficacy of different statin drugs against numerous cancers, such as breast (NCT00807950, NCT05550415), gastric (NCT01099085, NCT03086291), colorectal (NCT01238094), and bladder (NCT02360618) cancer, and these results support the development of new approaches, engaging statins in cancer management in the future [18].

Several types of nanocarrier systems, including liposomes, micelles, dendrimers, polymeric nanoparticles, and solid lipid nanoparticles, have been extensively explored in cancer therapy owing to their potential to improve drug solubility, enhance bioavailability, and minimize side effects [1]. Associated with these traditional systems, the chitosan-coated cubosomes used in our study offer additional benefits, such as better mucoadhesive properties, enhanced permeability across the intestinal lining, and pH-responsive drug release, making them especially suitable for oral delivery in colorectal cancer treatment. Standard chemotherapy for CRC often involves drugs such as 5-fluorouracil (5-FU), oxaliplatin, irinotecan, capecitabine, and leucovorin [19,20]. While effective, these drugs can lead to significant systemic toxicity and resistance over time. Our approach combines the advantages of a repurposed drug with a modern nanocarrier design to potentially overcome these challenges and offer a more targeted and effective treatment option.

Atorvastatin effectively downregulates the COX-2/PGE2/β-catenin signaling pathway, inducing cell cycle arrest at the G0/G1 phase, inhibiting the proliferation of CRC cancer cells, and inducing apoptosis [21,22]. Atorvastatin shows rapid absorption after oral administration with a peak plasma concentration at 1–2 h. Yet, its oral bioavailability is only 14% due to extensive first-pass metabolism by cytochrome P450 3A4 and is highly plasma protein-bound (over 98%) [23]. Loading ATR into nanocarriers is a suitable solution to benefit from the drug’s anticancer merits and to overcome its bioavailability problem [24]. Nanocarriers have demonstrated many advantages that captured researchers’ attention, whether enhancing the poor aqueous solubility of drugs, improving drugs’ oral bioavailability, or their ability to encapsulate hydrophobic and hydrophilic drugs for dual effects [25]. In addition, nanocarriers loaded with anticancer agents could tackle delivery-related problems and deliver anticancer agents to the intended tumor sites while evading unwanted side effects by regulating their pH, temperature, enzyme responsiveness, and tumor microenvironment [26]. Thus, chemotherapeutic agents encapsulated into nanocarriers could be delivered to tumors more precisely through efficient encapsulation and delivery.

Furthermore, our newest investigations presented novel zein-based nanoparticles (NPs) as an innovative approach for delivering repurposed drugs such as albendazole (ALB) to combat colorectal cancer (CRC). The optimized NPs exhibited excellent physicochemical characteristics, controlled pH-sensitive drug release, and improved anticancer efficacy by significantly lowering the IC50 in HCT116 cells. These promising results establish zein NPs as a potential nanocarrier system paving the way for enhancing CRC treatment strategies [27].

Likewise, liquid crystalline lipid nanoparticles are bicontinuous cubic nanosystems that have numerous advantages that aid in targeted drug delivery, including small size, biocompatibility, selective cell targeting due to the proteins and lipids present in their membrane, the ability to cross biological barriers and the gastrointestinal tract, and the ability to encapsulate both lipophilic and hydrophilic drugs [28,29]. Cubosomes are distinct, nanostructured particles characterized by an internal bicontinuous cubic liquid crystalline phase, representing a specific subclass of liquid crystalline nanoparticles; thus, the terms are used interchangeably throughout this study to describe these nanocarriers.

Drugs encapsulated in nanoparticles are masked from their surroundings and thus can be transported to specific sites, depending on their surface properties. Chitosan nanoparticles are gaining special attention due to their potential characteristics, such as mucoadhesive properties, positive surface charge, and ability to open tight junctions. Chitosan is a derivative of the natural polysaccharide chitin and the second most abundant polysaccharide in the world after cellulose. Chitosan is known for its biocompatibility, biodegradability, antibacterial effect, and mucoadhesion and is widely used in food, cosmetics, fabrics, and biomedical applications [30]. Chitosan can interact electrostatically with negatively charged molecules, such as cells, nanoparticles, lipids, drugs, and polymers, because of the functional amino groups on its surface. This polysaccharide has been extensively used as a surface-coating material for nanoparticles. It improves mucoadhesion and retention in the intestinal epithelium, enhances the stability of the loaded drug and cellular uptake, and prolongs the drug release time [31].

**Figure 1:**
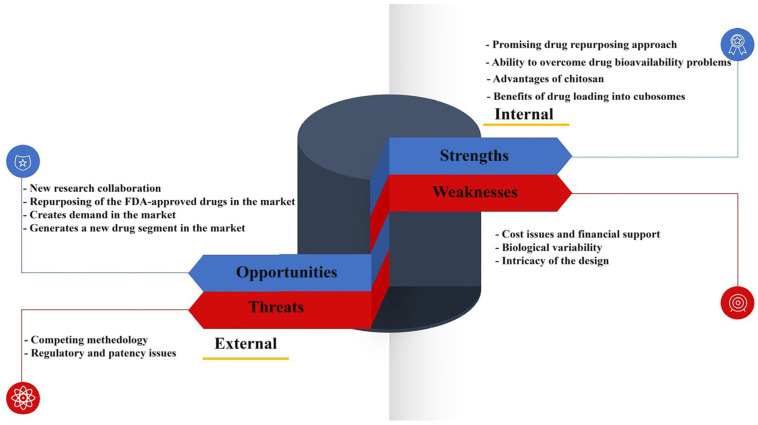
The SWOT analysis summarizes the strengths, weaknesses, opportunities, and threats linked with the research study on the formulated atorvastatin-loaded chitosan-coated cubosomes.

In this research attempt, we aimed to design and optimize through Box–Behnken design atorvastatin-loaded chitosan-coated cubosomes as an oral agent for treating CRC. Following this, the formulations were subjected to a comprehensive physicochemical characterization, and their effects on the growth and viability of HCT116 human CRC cells were assayed. The SWOT analysis illustrated in Figure 1 outlines the possible strengths, weaknesses, opportunities, and threats associated with our research investigation on the formulated atorvastatin-loaded chitosan-coated cubosomes for their anticancer activity on HCT-116 colon cancer cells.

## 2. Materials and Methods

### 2.1. Materials

Chitosan (CAS number: 9012-76-4, medium molecular weight “310–375 KDa”, Poloxamer 407^®^ (P407, Poly (ethylene glycol)-block-poly (propylene glycol)-block-poly (ethylene glycol)) and polyvinyl alcohol (PVA, Mw 89,000–98,000, 99+% hydrolyzed) were purchased from Sigma-Aldrich, Steinheim, Switzerland. Glyceryl monooleate (GMO) was kindly donated by Gattefosse Co. (Lyon, France), and atorvastatin calcium was generously gifted from Benta Pharma Industries—BPI, Lebanon. The HCT116 cell line was obtained from the American Tissue Culture Collection, ATCC, and Manassas, VA, USA. All other reagents and solvents used were of analytical grade.

### 2.2. Methods

This study involved three main experimental phases: the optimization of the preparation conditions of ATR-loaded cubosomes via the Box–Behnken design, followed by the formulation of these cubosomes (by the solvent dilution method) and their physicochemical characterization. Afterward, the optimized formula was coated with chitosan (0.5% *w*/*v*) solution. Finally, the optimized cubosomes underwent different evaluation tests and cellular assessments.

#### 2.2.1. Experimental Design

A Box–Behnken experimental design (2-factor, 3-level) was constructed using DESIGN EXPERT^®^ software, version 11, Stat-Ease Inc., Minneapolis, MN, USA. The effect of two independent variables, namely, the weight of P 407 used (mg) (A) and the concentration of PVA (B) on the ATR-loaded cubosomes particle size (nm) (Y1), uniformity index (Y2), and zeta potential (mV) (Y3) were studied (Table 1).

As mentioned above, the influence of the independent variables on the responses was investigated through mathematical equations, contour plots, and surface plots. An analysis of variance (ANOVA) was applied to evaluate the results, and the final equations were shortened to include only the significant parameters (*p*-value < 0.05). The best-fitting model was selected based on the adequate precision ratio, the predicted R^2^, and the software that generated the representative equations. Desirability was also calculated to select the optimized formula, which was subjected to further evaluation.

#### 2.2.2. Preparation of Blank and Atorvastatin-Loaded Liquid Crystalline Lipid Nanoparticles

**Figure 2:**
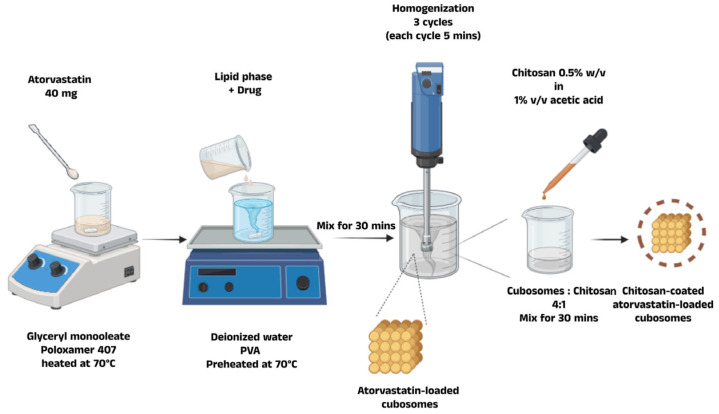
Schematic diagram representing the preparation of chitosan-coated atorvastatin-loaded liquid crystalline lipid nanoparticles.

For blank cubosomal dispersion, the selected GMO and poloxamer 407 weights were melted and stirred at 70 °C until both components were utterly homogenous. The molten solution was added dropwise to 30 mL of deionized water containing PVA heated to the same temperature (70 °C) and mixed for 30 min. This was followed by homogenization using a high-speed homogenizer (Shengwin, China 2000–18,000 rpm) for three cycles of 5 min each to achieve a homogenous state (Figure 2). The mixture was then equilibrated at room temperature for 48 h to obtain the cubic nanostructures. The drug-loaded cubosomes were prepared by dissolving 40 mg of ATR in the lipid mixture before adding the aqueous phase. The remaining process followed the same steps described for blank cubosome preparation [32]. The formulations were stored in sealed glass vials at 4 °C until required [33].

#### 2.2.3. Preparation of Chitosan-Coated Atorvastatin-Loaded Cubosomes

Chitosan-coated ATR-loaded cubosomes were prepared using 0.5% *w*/*v* chitosan solution. Different cubosomes to chitosan ratios were tested to select the optimal coating ratio based on the physicochemical properties of the optimized formulation, such as the smallest particle size, optimal uniformity index, and zeta potential. Chitosan solution was added dropwise to the cubosomal dispersion and mixed for 30 min [34].

### 2.3. Characterization of the Uncoated Atorvastatin-Loaded Cubosomes and the Chitosan-Coated Atorvastatin-Loaded Cubosomes

i. Physicochemical properties

The particle size, zeta potential, and PDI of cubosomal dispersions were determined by dynamic light scattering using Zeta Sizer 2000 (Malvern Instruments, Malvern, Worcestershire, UK) [35]. Samples were diluted with deionized water and measured in triplicate [36].

ii. Drug content and entrapment efficiency

The content of ATR-loaded cubosomal formulations was measured after dissolving the cubosomes in ethanol at a ratio of 1:50 *v*/*v*, followed by vortex mixing to ensure complete lysis of the vesicles [37]. The drug concentration was measured at the predetermined λmax (246 nm) using a UV spectrophotometer (Jasco V-730 spectrophotometer, JASCO UK Limited, W. Yorkshire, UK).

The following equation was used to compute the drug content in the cubosomes:

(1) $$Drug\;content\;\%=\frac{Actual\;ATR\;content}{Theoretical\;ATR\;content}\times 100$$

To determine the entrapment efficiency (EE), the specified amount of the loaded cubosome was centrifuged for 40 min at 20,000× *g* rpm at 4 °C using a high-speed centrifuge (Sigma—4L42, Sigma Laboratory Centrifuges, Osteride am Harz, Germany). The supernatant was removed, filtered, and diluted with ethanol to quantify the amount of free ATR spectrophotometrically at 246 nm [38,39].

The entrapment efficiency (EE) was calculated as follows:

(2) $$Encapsulation\;efficiency\;\%=\frac{Total\;amount\;of\;drug-Free\;drug}{Total\;amount\;of\;drug}\times 100\;$$

iii. Fourier transform infrared spectroscopy compatibility study

Fourier transform infrared spectroscopy (FTIR) was used to evaluate ATR’s state and detect the possible interaction between the drug and the cubosomal components. FTIR spectroscopy was recorded for ATR, physical mixture, and the prepared chitosan-coated ATR-loaded cubosomes at room temperature (Bruker Vector 42, Billerica, MA, USA). Dry potassium bromide was mixed with the samples, which were then compressed into a disc and scanned over a range of 2000–4000 cm^−1^ [40].

### 2.4. In Vitro Release of Atorvastatin from the Chitosan-Coated Atorvastatin-Loaded Liquid Crystalline Lipid Nanoparticles

Drug release studies were conducted using the United States Pharmacopoeia Dissolution Type II apparatus, employing the dialysis bag method at different pH values to stimulate gastrointestinal tract (GIT) conditions. Chitosan-coated ATR-loaded cubosomes equivalent to 40 mg of ATR were transferred to a dialysis bag (MWCO 12–14 kDa) secured at both ends and compared to commercially available ATR tablets. Before conducting the in vitro drug release studies, the solubility of atorvastatin was determined in relevant dissolution media (pH 1.2, 4.5, 6.8, and 7.4). This evaluation confirmed the selection of media and provided critical data for assessing sink conditions, particularly given the pH-dependent solubility of the drug [41,42]. The experiment was performed in 200 mL of dissolution media of four different pHs, 0.1 N hydrochloric acid (pH 1.2) for simulated gastric fluid (SGF), phosphate buffer of pH 4.5 mixture of SGF and simulated intestinal fluid (SIF), phosphate buffer 7.4 (SIF), and phosphate buffer of pH 6.8 and simulated colonic fluid (SCF). The procedure was carried out at a constant 37 °C ± 1 temperature under sink conditions. The incubation times of the samples in the media mentioned above were selected to represent the gradual pH change in the digestive tract, which varies between the stomach (pH 1.2) and the colon (pH 6.8), as shown in Table 2 [43]. An aliquot was removed and analyzed spectrophotometrically for drug content and replaced with fresh media to maintain sink conditions at different time intervals, with suitable fresh media as a blank. To determine the mechanism of ATR release and release kinetics from the prepared cubosomes DDSolver, an Excel add-in was used through a variety of release models, including zero-order, first-order, Higuchi, Hixson–Crowell, and Korsmeyer–Peppas models [36].

### 2.5. Analytical Method Validation

The validation of the analytical method ensures that the developed process is acceptable and reliable for its projected purpose. The UV–vis spectrophotometric method used for atorvastatin quantification was validated following the International Council for Harmonisation (ICH) guideline Q2(R2) on “Validation of Analytical Procedures” [44]. The validation parameters included linearity, expressed as the correlation coefficient, repeatability (intra-assay precision), expressed as the % relative standard deviation (%RSD), limit of detection (LOD), and limit of quantification (LOQ) [45]. All absorbance measurements were performed in triplicate and presented as mean ± SD.

(3) $$LOD=\frac{3.3\times \sigma }{S}$$

(4) $$LOQ=\frac{10\times \sigma }{S}$$

(5) $$\%\;RSD=\frac{\sigma }{Mean}\times 100$$

where

σ = the standard deviation of the response
S = the slope of the calibration curve

### 2.6. Cell Lines and Cell Culture Conditions

HCT116 wild-type for p53 (tumor suppressor gene) cells were grown in RPMI 1640 (Lonza, Basel, Switzerland) medium, 10% fetal bovine serum, 1% penicillin–streptomycin antibiotics, and 1% sodium pyruvate (Sigma-Aldrich, St. Louis, MO, USA). Cells were incubated in a humidified incubator (95% air, 5% CO_2_) at 37 °C [46]. Atorvastatin solution, unloaded-uncoated cubosomes (-C-D), ATR-loaded chitosan-coated cubosomes (+C+D), uncoated ATR-loaded cubosomes (-C+D), and unloaded chitosan-coated cubosomes (+C-D) were tested on HCT116 cell lines for their ability to inhibit cell proliferation and reduce cell viability [47].

i. Sulforhodamine B Assay

A 96-well plate with a seeding density of 5000 cells per well was used to culture the cells for 24, 48, and 72 h. Serial dilutions of the tested samples were applied to the designated wells and incubated for the mentioned time points. After incubation, cells were fixed using 50% *w*/*v* trichloroacetic acid solution, washed, and incubated with Sulforhodamine B (SRB) salt (0.4% *w*/*v*) to be stained at room temperature for one hour. Following the plates, they were washed four times with 1% acetic acid (*v*/*v*) solution and left to air-dry for 30 min. Bound SRB was solubilized with 10 mM Tris base solution pH = 10.5. The plates were read at an absorbance of 595 nm using the ELISA Multiskan Ex microplate reader (Thermo Fisher Scientific, Waltham, MA, USA) [48].

ii. Trypan blue cell viability assay

Aiming to confirm cell cytotoxicity results, the trypan blue assay was performed. The trypan blue assay is applied to calculate the live and dead cells in a cell suspension because cells with intact cell membranes (alive) prohibit the entry of trypan blue dye (appears light in color). In contrast, dead cells with ruptured cell membranes accumulate the dye (appear blue) [49]. Cells with a density of 25,000 cells/well were seeded in 9 well plates. After 24, 48, and 72 h of incubation, cells were treated with serial ATR solution, unloaded-uncoated cubosomes, ATR-loaded chitosan-coated cubosomes, uncoated ATR-loaded cubosomes, and unloaded chitosan-coated cubosomes dilutions. Media, including floating dead cells, were collected, and adherent living cells were detached by trypsin-EDTA and added to the media. From a 500 µL cell suspension, 50 µL was mixed with 50 µL of trypan blue, and then live/dead cells were counted using a hemocytometer [48].

### 2.7. Stability Study

Samples of loaded cubosomal formulations were stored in tightly closed glass vials at 4 °C and −8 °C for 3 months. The samples were withdrawn monthly, diluted with deionized water, and assessed for particle size, zeta potential, and polydispersity index (PDI). All reported data are the mean of three separate measurements [50].

These storage conditions were nominated based on their significance to biological and temperature-sensitive formulations. Former studies have established that lipid-based nanocarriers exhibit superior physicochemical stability and conserve structural integrity when stored at refrigerated or frozen temperatures. In contrast, room-temperature storage often leads to particle degradation and reduced functional performance [51,52].

### 2.8. Statistical Analysis

The results were expressed as mean ± SD, and the statistical analysis of the obtained data was conducted using an analysis of variance (Statistical Package for the Social Sciences (SPSS) version 22 (IBM, New York, NY, USA). A probability level of *p* < 0.05 was considered significant.

## 3. Results and Discussion

### 3.1. Analysis of Box–Behnken Experimental Design Results

The particle size, polydispersity index, and zeta potential for the 12 formulated ATR-loaded liquid crystalline lipid nanoparticles are tabulated in Table 3. Aliases in any factorial design are not favored, as they reflect the inability to estimate the effect of the variables under study. If these variables are aliased, they may cause a combined effect on the investigated responses. The model applied for this analysis showed no desired aliases.

For an optimal balanced design, the standard errors of the studied variables should be similar and less than one (Table 4). The lower the standard error, the more applicable the model is for analysis. Variance inflation factor (VIF) measures the level of collinearity in regression analysis. Detecting multicollinearity affects the statistical significance of the independent variables. A large VIF above 10 indicates a high collinear relation between the variables, indicating that the coefficients are poorly estimated; thus, the model should be adjusted. The ideal VIF value is 1.0, as in our design model. Similarly, Rᵢ^2^ should be zero, as a high Rᵢ^2^ means that the terms are correlated, possibly leading to poor statistical models [53].

**Figure 3:**
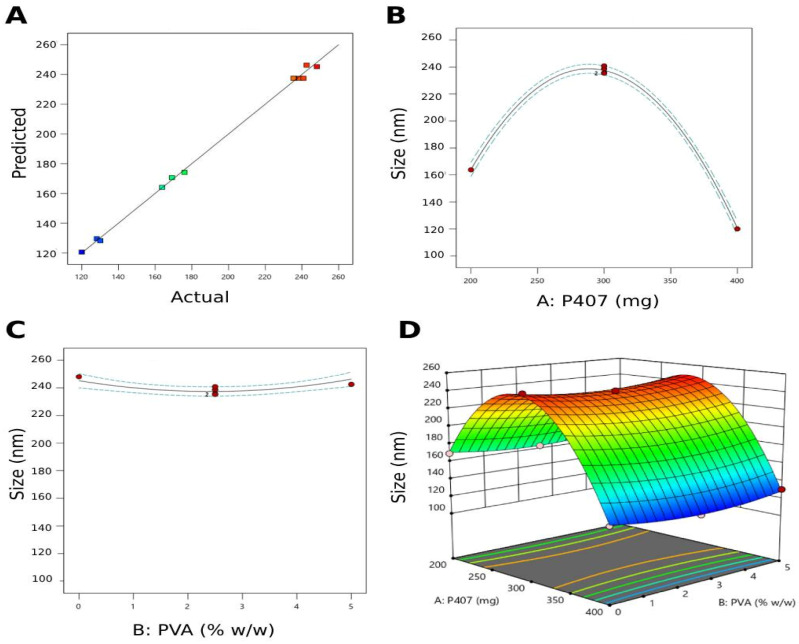
Graphical representation of the studied variables and the investigated particle size as a response in the applied Box–Behnken design for the atorvastatin-loaded liquid crystalline lipid nanoparticles. (**A**) Predicted versus actual diagram. (**B**) Main effect diagram of poloxamer 407 weight used (mg); (**C**) main effect diagram of PVA (% *w*/*w*); (**D**) 3D Surface plot representing the effect of poloxamer 407 and PVA concentrations on cubosome particle size.

The predicted and adjusted R^2^ values should be within 0.2 of each other for a model to have a good acceptance. There was a good agreement between both R^2^ values for the studied responses. The adequate precision value must be above 4, as it designates a good signal-to-noise ratio; thus, the model can be used to navigate the design space [54]. The value for adequate precision was higher than 4 for the examined responses (Table 5).

i. Particle size:

The quadratic model best fits the particle size data with a *p*-value < 0.0001 (Table 5). The model F-value of 632.13 implies that the model is significant. There is only a 0.01% chance that an F-value of this magnitude could occur due to noise. The model’s validity is supported by a non-significant lack of fit (*p*-value 1.69), which indicates that the model can fit the data. The predicted and actual particle size values are plotted in Figure 3A. All the points were located on the same line, displaying a good agreement between these values and supporting the validity of the design model.

The influence of formulation variables on particle size is presented in the following equation:

(6) $$Y_{1}=+237.45-21.76A+0.5B-1.22AB-95.1A^{2}+8.32B^{2}$$

where Y_1_ is the particle size, A is the weight of poloxamer 407 (mg), and B is the % *w*/*w* of PVA.

From the above equation, the concentration of P407 is observed to exert the highest effect on the particle size, showing a negative correlation. As the weight of P407 used increases, the particle size decreases, since poloxamer provides steric stabilization to cubosomes by anchoring its polypropylene oxide blocks in the polar region or at the surface of the GMO bilayer, preventing their coalescence and agglomeration [55]. The particle size of the voriconazole-loaded cubosomes formulated by Alhakamy et al. was negatively affected by the increased concentration of poloxamer, and the authors attributed this effect to the ability of the non-ionic surfactant to reduce the interfacial tension between the lipid matrix and the surrounding aqueous phase, resulting in smaller vesicles [56].

This is also presented in Figure 3B,C, where a significant decrease in particle size resulted from an increased concentration of P407. In contrast, the PVA concentration was ineffective in inducing any substantial change in cubosome particle size. The 3D surface plot supported the above-mentioned remarkable effect of P407 weight on the particle size of the prepared cubosomes, while that of PVA was negligible (Figure 3D).

ii. Particle size distribution analysis

**Figure 4:**
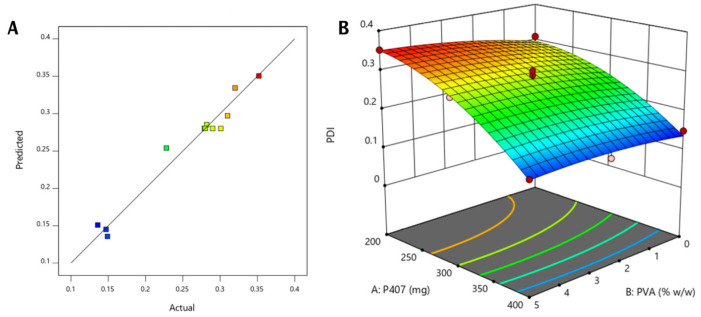
Graphical representation of the studied variables and the investigated PDI as a response of the applied Box–Behnken design for the atorvastatin-loaded liquid crystalline lipid nanoparticles. (**A**) Predicted versus actual diagram. (**B**) Three-dimensional surface plot representing the effects of the weight of poloxamer 407 used (mg) and the concentration of PVA (% *w*/*w*) on the cubosome particle size distribution reflected by polydispersity index.

The effect of the independent variables, specifically, the percentage of PVA and the weight of poloxamer 407 on the particle size distribution indicated by the PDI, was best fitted in a quadratic model (*p*-value 0.0002) with a significant model F-value (34.74), with only a 0.02% probability of noise in the design (Table 5). The model validity is braced by a non-significant lack of fit (*p*-value 5.29). The predicted and actual PDI values plotted were found to be positioned on the same line, displaying a good agreement and supporting the model’s validity (Figure 4A).

The influence of formulation variables (weight of P407 and % *w*/*w* of PVA) on cubosomes size uniformity is presented in the following equation:

(7) $$Y_{2}=0.280-0.092A+0.0156B-0.011AB-0.0375A^{2}-0.0105B^{2}$$

In Equation (7), Y^2^ is the polydispersity index, A is the poloxamer 407 (mg) weight, and B is the % *w*/*w* of PVA.

The PDI shows the width of the overall particle size distribution, whereas a smaller PDI value reflects a monodisperse system [54]. According to Equation (7), both independent variables had minimal PDI effects. Increasing P407 from 200 to 400 mg caused a significant decrease in the PDI, while increasing the PVA concentration caused a minor increase in this value, yet all the values were less than one. Consequently, it was believed that the combined effect of poloxamer 407 and PVA supported the steric stability of the cubosomal system, where PVA acted as a stabilizer, and P407 promoted the stability of the vesicles by decreasing the interfacial tension between GMO and water, in addition to achieving steric stabilization, which leads to uniform, non-aggregated cubosomes [57,58]. The effect of P407 weight (mg) and PVA concentration (% *w*/*w*) was also reflected in the 3D surface plot, which demonstrated the downward trend in the graph upon increasing P407 concentration, and an almost parallel line against PVA, indicating its minimal effect (Figure 4B). It was proved that higher amounts of poloxamers provided a competent surface coverage during the formation of these cubosomes due to the reduced surface tension of the oil droplets, hence permitting the dispersion of the water phase and stabilizing the interface, creating a homogenous and uniform emulsion that formed nanosized cubic-phase nanoparticles with a small PDI upon cooling down [57].

iii. Zeta potential

**Figure 5:**
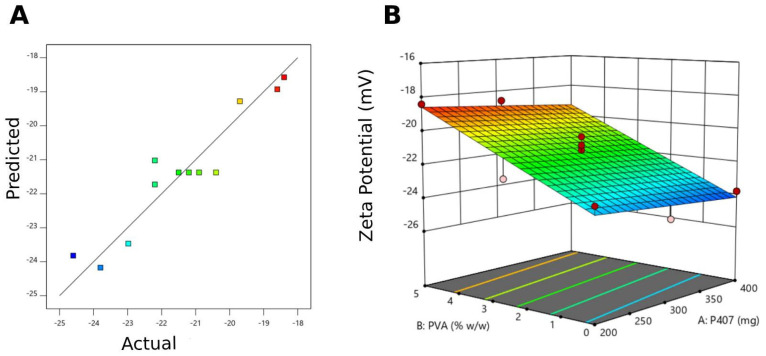
Graphical representation of the studied variables and the investigated zeta potential, as a response of the applied Box–Behnken design for the atorvastatin-loaded liquid crystalline lipid nanoparticles. (**A**) Predicted versus actual diagram. (**B**) Three-dimensional surface plot representing the effects of poloxamer 407 and polyvinyl alcohol (PVA) on zeta potential values.

Concerning the effects of the poloxamer 407 weight and PVA concentration on the surface charge of the liquid crystalline lipid nanoparticles, the best-fit model was linear (*p*-value < 0.0001), with a significant model F-value (39.93) and only a 0.01% likelihood that this F-value resulted from noise in the design (Table 5). The model validity is assisted by a non-significant lack of fit. The predicted values, plotted against actual zeta potential values exhibited a linear relationship, indicating a mutual agreement between the theoretical and the experimental values and supporting the validity of the design model (Figure 5A). The predicted R^2^ of 0.8209 is in reasonable agreement with the adjusted R^2^ of 0.8762 (difference < 0.2), and the adequate precision that measures the signal-to-noise ratio (16.530 > 4) indicates that this linear mathematical model can be used to navigate the design space.

The linear equation representing the effect of independent variables on the zeta potential value is as follows:

(8) $$Y_{3}=-21.373-0.3533A+2.4467$$

where Y3 is the zeta potential value, A is the poloxamer 407 (mg) weight, and B is the concentration of PVA (%*w*/*w*).

Zeta potential is a key parameter used to determine the long-term stability of colloidal dispersions, where a high absolute value is desired for optimal stability. The zeta potential value displays the magnitude of electrical repulsion forces between the particles, and the high repulsion forces prevent particles from aggregation and coalescence. The zeta potential values of the prepared cubosomes ranged from −18.4 ± 2.70 mV to −24.6 ± 1.58 mV, as shown in Table 3, which indicates good stability of the prepared cubic nanoparticles. Similar results were attained by Eldeeb et al. when formulating brimonidine tartrate cubosomes using GMO, PVA, and P407, where the negative zeta potential values ranged from −31.9 mV to −24.6 mV, reflecting the magnitude of electrical repulsion forces between the particles, and indicating good stability of the prepared formulations [37]. The negative charge of the cubosomes is attributed to the ionization of the carboxylic end group of the free fatty acid (oleic acid) present in GMO and the interaction between the poloxamer 407 hydroxyl group and the aqueous medium [59]. In addition, the concentration of PVA has a significant positive effect (Figure 5B) on increasing ZP due to the PVA hydroxyl groups that are adsorbed at the particle surface, making it more accessible for ionization [60].

The optimized formula provided by the software, composed of 400 mg poloxamer 407 and PVA (2.73% *w*/*w* of GMO), with a desirability of 0.974, was selected for the analysis. There was a non-significant difference between the size, PDI, and zeta potential values, indicating a good fit of the model, as illustrated in Table 6.

### 3.2. Chitosan-Coated Atorvastatin-Loaded Cubosomal Formulation

**Figure 6:**
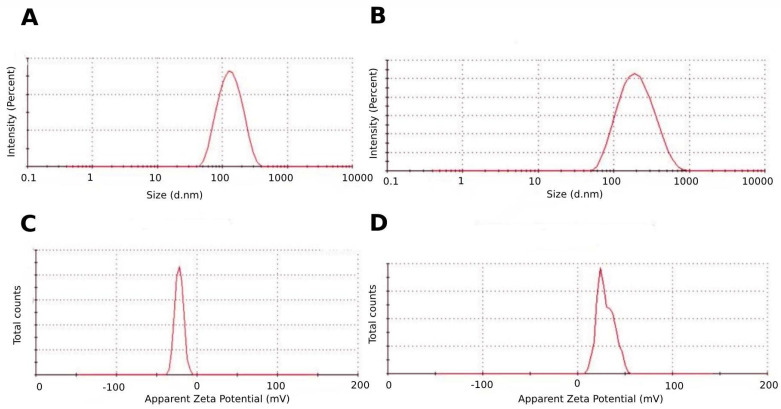
Particle size and zeta potential of the uncoated atorvastatin-loaded cubosomes (**A**,**C**); (**B**,**D**) represent the particle size distribution and zeta potential of the optimized chitosan-coated atorvastatin-loaded cubosomes, prepared at a cubosome-to-chitosan ratio of 4:1 (*v*/*v*).

Various coated formulations were prepared utilizing different ratios of cubosomes and chitosan and were then evaluated to select the optimal coated formula with a positive zeta potential, as well as uniform particles with smaller sizes. It was found that a cubosome-to-chitosan volume ratio of 4:1 (*v*/*v*) was adequate, and the chitosan-coated optimum cubosomal formulation had an average particle size, PDI, and zeta potential of 169.3 ± 4.14 nm, 0.245 ± 0.015, and 29.7 ± 0.814 mV, respectively (Figure 6). The surface charge of the loaded cubosomal formulation shifted to a high positive value due to the electrostatic adsorption of positively charged chitosan onto negatively charged cubosomes [55,61]. The increase in the mean particle size validates the successful adsorption of a thick chitosan coat on the surface of cubosomes, and the same applies to broadened size distribution [62].

### 3.3. Physicochemical Characterization of the Uncoated and Coated Atorvastatin-Loaded Cubosomes

The entrapment efficiency and drug content of the cubosomal formulation are 92.078 ± 2.46% and 64.51 ± 1.58%, respectively, indicating that ATR was efficiently entrapped inside the cubosomes. This can be attributed to the robust attraction between the poorly soluble drug and the hydrophobic domain of the cubic-phase bilayer, permitting complete incorporation [63]. Lipid-based nanocarriers containing surface-active agents amplify drug-loading efficiency by stabilizing the lipid system and enhancing drug solubility [25]. The presence of poloxamer 407 alongside glyceryl monooleate created a highly lipophilic core, allowing the formulated cubosomes to encapsulate more ATR. Incorporating the polypropylene oxide groups of P407 in the polar region at the surface of the GMO created steric stabilization, allowing a higher amount of drug to be loaded into the formed bilayer. Alamoudi et al. reported a similar outcome upon formulating 5-fluorouracil-loaded cubosomes using glyceryl monooleate and poloxamer 407; drug entrapment ranged between 89–96% in the prepared formulations, and this was attributed to the presence of poloxamer 407, which increased the entrapment of drugs into a hydrophobic carrier such as GMO [64].

**Figure 7:**
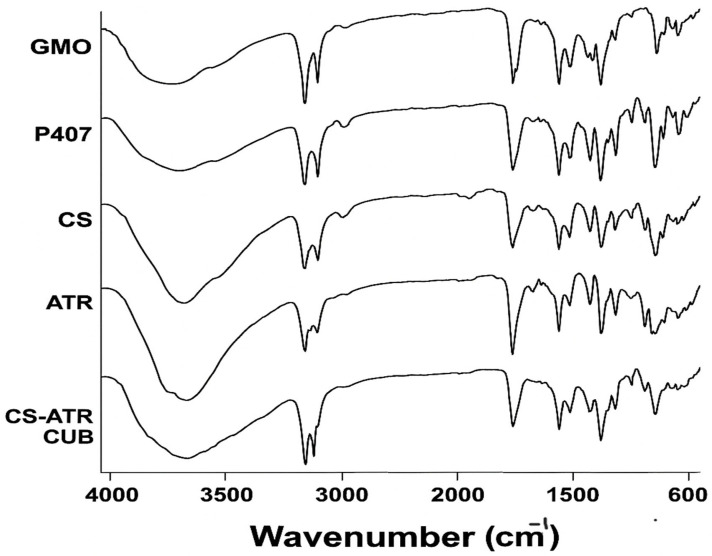
Fourier transform infrared (FTIR) spectra of pure glyceryl monooleate (GMO), poloxamer 407 (P407), chitosan (CS), atorvastatin (ATR), and chitosan-coated atorvastatin-loaded cubosomes (CS-ATR-CUB).

The FTIR spectra of chitosan-coated ATR-loaded cubosomes, raw ATR, the components of the cubosomes (GMO and P407), and chitosan are visualized in Figure 7. The FTIR analysis was applied to rule out any interaction between the cubosomal components, the coating material, and the drug [35]. Atorvastatin shows intense absorption bands at 3365 cm^−1^ (O-H stretch), 3243 cm^−1^ (aromatic N-H stretch), 1650 cm^−1^ (aromatic C=O stretch), 1316 cm^−1^ (C-O stretch of carboxylate group), and 695 cm^−1^ (aromatic out-of-plane bend). These analyzed peaks were also represented by the previously fabricated ATR-loaded solid lipid nanoparticles [65]. The characteristic absorption bands of chitosan are presented particularly at 3423 cm^−1^ (OH-group), 2882 cm^−1^ (C-H stretching vibration), 1654 cm^−1^ (C=O of amide group), 1562–1421 cm^−1^ (C-H of acetamide and N-H of amide II), and 1153–1076 (C-N and C-O stretching vibrations). These representable peaks of chitosan were mutual to those detected when analyzing curcumin-loaded eudragit-decorated chitosan nanoparticles and chitosan-coated coconut oil-based cubosomes [61,66]. Similarly, the FTIR spectra revealed characteristic bands at 3422 cm^−1^ (-OH group), 2924–2853 cm^−1^ (CH2 stretching), and 1741 cm^−1^ (C=O) for GMO; and 3482 cm^−1^ (-OH group), 2970 cm^−1^ (C-H group) and 1111 cm^−1^ (C-O group) for poloxamer 407, as previously reported [67,68]. In the FTIR spectra of chitosan-coated ATR-loaded cubosomes, some ATR peaks were not detected, representing the effective entrapment of the drug within the lipid matrix. In contrast, other characteristic peaks of the cubosomal excipients were still present, indicating the absence of any interaction between GMO, P407, and ATR. In addition, no new peaks were created, and the essential bands of chitosan were still present after designating the effectual coating of the cubosomes. These characterization results follow the previously reported work by Azzazy et al., where the FTIR spectra of chitosan-coated alkaloid-loaded poly (lactic-co-glycolic acid, PLGA) nanoparticles presented characteristic peaks of PLGA at 1076 and 1721 cm^−1^ and of chitosan at 3277 and 3384 cm^−1^, as well as the significant bands of the alkaloid, with no noticeable shift, suggesting successful drug entrapment and the efficient coating with chitosan [69].

### 3.4. In Vitro Release and Kinetic Studies of Chitosan-Coated Atorvastatin-Loaded Liquid Crystalline Lipid Nanoparticles

**Figure 8:**
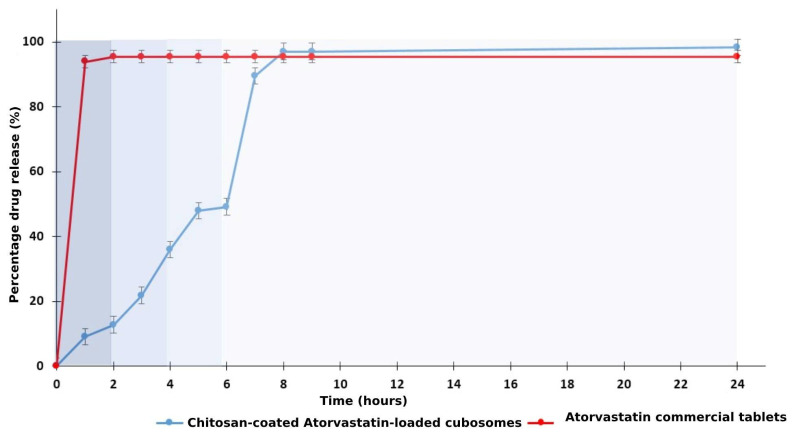
In vitro release graph of chitosan-coated atorvastatin-loaded cubosomes, compared with atorvastatin commercial oral tablet in different pH media to represent the gradual pH change in the digestive tract, which varies between the stomach (pH 1.2, 0–2 h), simulated gastric fluid+ simulated intestinal fluid (pH 4.5, 2–4 h), simulated intestinal fluid (pH 7.4, 4–6 h) and simulated colon fluid (at pH 6.8 for 6 h).

The in vitro release of chitosan-coated ATR-loaded cubosomes was studied in comparison with that of the commercial oral tablet to point out the efficiency of the coated cubosomal formulation in delivering the drug to the colon without being affected by the change in pH through the GIT (Figure 8). For the commercial tablet, almost all of the ATR was released within the first hour of this study (85.55 ± 5.58%). The graph illustrates a sustained, controlled release of the drug from the chitosan-coated cubosomes. In the first two hours in the SGF, a meager amount of ATR was released from the cubosomes (9.83 ± 0.24%). Next, when the cubosomes reached a pH of 4.5 (2–4 h), the slow release of ATR increased to 35.94 ± 0.7% at the end of the 4 h. The release of ATR significantly improved in pH 7.4 medium (SIF) to reach 49.24 ± 0.39% at the end of 6 h. Upon reaching pH 6.8 (SCF), a remarkable increase in the release was established, where it elevated to 89.64 ± 1.04% at 7 h and 98.42 ± 1.58% at the end of the 24 h experiment. The presence of chitosan created a smart pH-dependent drug release, wherein the acidic medium, the electrostatic attraction between protonated amine groups, and water allowed slight matrix erosion and drug release. At higher pH levels, the swelling of the chitosan-coated cubosomes was greater, allowing the dissolution to penetrate deeper into the polymer matrix, thereby facilitating efficient and quicker drug diffusion out of the cubosomes [70]. Due to the protonation amino groups (-NH^3+^), chitosan (CS) is highly positively charged at low pH. The interaction of H+ ions (at acidic pH) with cations on the CS surface limits the polymer hydrolysis, and this clarifies the reason why ATR release from the cubosomes occurred more rapidly in a high pH environment than in the acidic one [71]. Similar results were recognized with chitosan/alginate/lovastatin nanoparticles, where the drug release rate from the nanoparticles was proportional to the increase in the solution pH in the order of pH 7.4 > pH 6.5 > pH 4.5 > pH 2.0. The slow release at low pH media was due to the repulsion between the hydrogen ions in the acid medium and the cations on the CS surface, indicating the suitability of this polymer for drug delivery in the intestine and colon [72].

The best-fit model for ATR release from the fabricated nanosized coated cubosomes was Korsmeyer–Peppas, with the highest R^2^ (0.9546) and the diffusion coefficient *n* = 0.216 (Table 7). The release may follow Fickian diffusion if the n value is less than or equal to 0.5 [73]. The Korsmeyer–Peppas model describes a diffusion-controlled release from matrix-type nanosystems, where ATR release occurs by the initiation of polymer relaxation of the nanoparticle in SIF, and then Fickian diffusion continues for the CS-coated cubosomes through the polymer matrix via diffusion, erosion, and matrix degradation, leading to drug release [70,74]. When exposed to aqueous media, CS hydrates to form a viscous layer, allowing the drug to be released from its matrix. A major regulator of this process is the diffusion of the drug throughout the swollen matrix, as well as the erosion of the swollen CS matrix. This mode of release is almost common when chitosan is used as a coating material, where the release of metronidazole from chitosan nanoparticles is rapid at first, governed by Fickian diffusion of the drug through the polymer network. Similarly, the release of 5-fluorouracil from chitosan nanoparticles followed the exact mechanism [75,76].

### 3.5. Validation of the Quantification Method

The UV–vis spectrophotometric method used to quantify atorvastatin was validated. A calibration curve (0.005–0.02 mg/mL) yielded an R^2^ value of 0.949, with the regression equation Abs = 48.556 × Conc. + 0.015.

The analytical method established suitable sensitivity and reliability for atorvastatin quantification, within the studied concentration range. The calculated limit of detection (LOD) of 0.0057 mg/mL and limit of quantification (LOQ) of 0.0172 mg/mL suggest that the method is capable of detecting and accurately quantifying even low concentrations of atorvastatin in formulation matrices.

The correlation coefficient (R^2^) of 0.949 reveals adequate linearity for early-stage analytical work, though minor variability at lower concentrations is noted. Furthermore, intra-day precision, expressed as %RSD, remained below 5.2% across most concentration levels, representing good repeatability. The slightly higher %RSD at the lowest concentration (12.84%) is predictable due to its closeness to the LOD and does not impact the method’s overall applicability for encapsulation efficiency and drug release quantification.

### 3.6. Sulforhodamine B and Trypan Blue Cell Viability Assays

Drug-loaded lipid-based systems have become a notable approach in therapeutic research. Amid this, monoolein-based nano-cubosomes have emerged as one of the most cost-effective and clinically promising technologies in disease diagnosis and treatment. Cubosomes possess a unique nanostructure of a three-dimensional folded curved bilayer of a lipophilic and hydrophilic domain to integrate water-soluble, oil-soluble, and amphiphilic substances [77]. In addition to being biocompatible and biodegradable, cubosomes can incorporate higher drug amounts than liposomes, as well as protect the drugs against physiological or chemical degradation [78].

Statins are potent inhibitors of the mevalonate/cholesterol biosynthetic pathway and are widely prescribed for preventing cardiovascular diseases. Studies have focused on applying different statin drugs based on their ability to inhibit the cholesterol biosynthetic pathway in tumor cells and thus provide potential benefits in controlling tumorigenesis and progression [79]. Among different statins, ATR is an effective, well-tolerated, synthetic, lipophilic, and inexpensive statin that has been investigated for its inhibitory effects on glioblastoma, prostate, colon, and breast cancer cells [80,81]. One of the strategies to potentiate the effect of ATR and overcome its solubility and poor oral bioavailability problem is loading the drug into nanocarriers. In this study, ATR was loaded into liquid crystalline lipid nanoparticles.

The sulforhodamine B (SRB) data showed a significant decrease in HCT116 cell viability in the ATR-treated group at concentrations 0.5, 1, and 5 µM on the second and third days of treatment. In comparison, in the uncoated ATR-loaded cubosomes, the reduction in cell viability was significant only as early as day one post-treatment at 5 µM (Figure S1, Supplementary Materials). These results agree with those attained by Xiao et al., where ATR inhibited HCT116 cell growth at concentrations between 5 and 8 µM [82]. On the other hand, Rao and Rao examined the effect of ATR on HT-29 and HCT116 CRC cell lines and presented a significant decrease in cell proliferation at a concentration of 12.5 μM; hence, this promising 2.5-fold reduction in the concentration is attributed to the encapsulation of atorvastatin in the liquid nanocrystalline nanoparticles [83].

As for the cells treated with chitosan-coated unloaded cubosomes, they showed a decrease in cell viability only at high concentrations of chitosan (5 µM) at days two and three post-treatment. The effect of a high chitosan concentration on cell viability was consistent with the findings of Abdelwahab et al., who observed a significant reduction in HCT116 cell viability at high concentrations (2.5, 5, and 10 µM) exhibited [84]. After analyzing the SRB data, it was concluded that ATR concentrations of 0.01, 1, and 5 µM would be used for the trypan blue assay to evaluate the effect of the studied formulations on cell viability.

**Figure 9:**
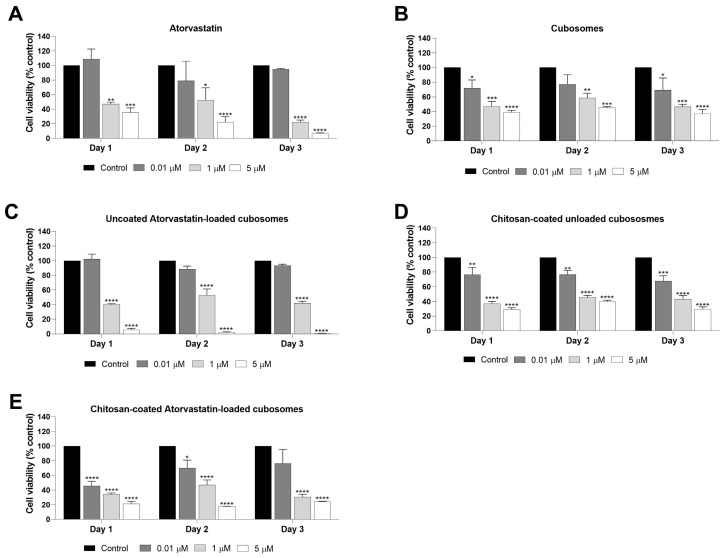
Effect of different treatments on the viability of colorectal cancer cells. HCT116 cells were seeded in a 24-well plate at a seeding density of 20,000 cell/well in triplicates. The cells were treated with the indicated concentrations of (**A**) atorvastatin, (**B**) cubosomes, (**C**) uncoated atorvastatin-loaded cubosomes, (**D**) chitosan-coated unloaded cubosomes, and (**E**) chitosan-coated atorvastatin-loaded cubosomes. The trypan blue dye exclusion assay results are expressed as a percentage of control and represent the average of three independent experiments ± SEM. The asterisks represent concentrations that showed a significant decrease in cell viability in treated versus control cells. Significance relative to the control is indicated by * *p* < 0.05, ** *p* < 0.01, *** *p* < 0.001, and **** *p* < 0.0001.

The influence of different treatments on HCT116 cell viability was further assayed by the trypan blue exclusion assay (Figure 9). The results showed the same trend as the SRB data. The ATR effect on cell viability was attained as early as day one post-treatment at concentrations 1 and 5 µM (Figure 9A). Upon loading ATR into cubosomes, the effect on cell viability was almost similar to that of the free drug, although it was more pronounced at 5 µM (Figure 9C). The difference between the effect of free drug and drug-loaded into cubosomes indicates that the drug’s anticancer activity has not been adversely affected by its loading onto the cubosomes. The uncoated ATR-loaded cubosomes showed a significant effect as early as day one post-treatment at concentrations 1 and 5 µM (*p*-value < 0.001 and *p*-value < 0.0001, respectively) and complete inhibition of viable cell count at a concentration of 5 µM (*p*-value < 0.0001), along with the presence of dead cells ~ 5 × 10^4^ cells (Supplementary Materials, Figure S2). Loading drugs into nanocarriers has always been effective in enhancing the drug’s effect against different cancer cells; for instance, loading ATR into polymeric micelles decreased its IC50 against HCT116 cells from 6.8 to 6.1 µg/mL at 48 h and from 9.4 to 3.4 µg/mL at 72 h [85]. Saber et al. accomplished analogous results where cisplatin inhibited 50% of HCT116 cell viability at a concentration of 15 µM, and upon loading cisplatin into nanocubosomes, the same effect was achieved, but at a lower concentration (9.6 µM) [86]. Likewise, loading 5-FU into cubosomes decreased its half-maximal inhibitory concentrations from 112.70 mg/mL for the free drug to 107.78 mg/mL for 5-FU cubosomal dispersion. In addition, the blank cubosomes exhibited a low effect on the human hepatoma HepG2 cell line at concentrations of 50 µg/mL and above [87].

Although there was a visible decrease in HCT116 cell viability in the group treated with unloaded cubosomes, this effect was mostly noticeable at high concentrations (1 and 5 µM) (Figure 9B). This cytotoxicity of cubosomes was also detected in other studies on different cancer cells and was considered insignificant when compared with the effect of cubosomes when loaded with drugs. Monooleate cubosomes were observed to have a cell death percentage of ≤20% at concentrations up to 125 μg/mL against PC-3, DU-145 (prostate cancer), AGS (gastric cancer), and PNT-1A (non-cancerous) [88]. Similarly, glyceryl monooleate cubosomes showed 40% toxicity at 100 and 75 µg/mL in Chinese Hamster Ovary cells, and they were non-toxic at a concentration of 100 µg/mL in the human alveolar basal epithelial cells [89]. In addition, the blank cubosomes assayed for their effect on HCT116 cell viability showed a decrease in the survival fraction of the cells at concentrations of 200 µg/mL and above [86]. In addition, blank cubosomes were toxic for human-derived HeLa cells when the GMO concentration reached 54 µg/mL or higher [90].

The appraisal of HCT116 cell viability, based on Figure 9E (chitosan-coated atorvastatin-loaded cubosomes) and Figure 9D (chitosan-coated unloaded cubosomes) reveals notable differences in cell viability trends. Both preparations displayed dose-dependent declines in cell viability upon increasing concentrations (0.01 μM, 1 μM, and 5 μM). However, the atorvastatin-loaded cubosomes in Figure 9E demonstrated considerably heightened cytotoxic effects compared to the unloaded cubosomes in Figure 9D, particularly at higher concentrations. This suggests that atorvastatin contributes substantially to the observed reduction in cell viability. Though the unloaded cubosomes indicated steady reductions in viability, the loaded formulation resulted in more significant decreases, with this effect remaining evident over the three days. The delivery of most of the nanocarriers, including chitin and chitosan, can follow either passive targeting or active targeting for drug delivery, and maintaining the effect of the drug upon loading is of great importance, as some drugs may lose their potential impact upon loading into nanocarriers, whether due to nanocarrier instability, premature degradation of the drug, or the inability of the drug to escape the nanocarrier, leading to a loss of therapeutic efficiency [91,92]. Though the uncoated cubosomes showed more effects on days two and three of the experiment at 5 µM compared to the coated ones, this may be attributed to the easier release of ATR in the case of the uncoated cubosomes. The ability of the uncoated cubosomes to deposit in the aqueous channels facilitates greater drug release, which may exert a superior in vitro effect on cells compared to the coated cubosomes. This was also in line with research by Zhang et al., who found that the poly-Ɛ-lysine-coated cisplatin and paclitaxel-loaded cubosomes showed significantly lower toxicity for the human hepatoma HepG2 cell line compared to the uncoated ones. It was concluded that coating cubosomes can also reduce their cytotoxicity [93]. Therefore, our findings indicate that chitosan-coated ATR-loaded cubosomes are promising liquid crystalline lipid nanocarriers for ATR administration against HCT116 human CRC cells. This highlights the potential of a drug repurposing approach in colorectal cancer management.

### 3.7. Stability Studies

Stability assessments were conducted at +4 °C and −8 °C to evaluate the preservation of the nanocarrier system under conditions pertinent to biological storage. This approach aligns with findings by Ball et al., who demonstrated that lipid nanoparticles exhibit superior stability and sustained efficacy when stored at refrigerated temperatures, with diminished performance observed at ambient conditions [94]. Ball et al. assayed the stability of lipid nanoparticles (LNPs) under various storage conditions, including refrigeration (2 °C), freezing (−20 °C), and room temperature. The findings indicate that LNPs preserved optimal stability and efficacy when stored at 2 °C for over 150 days, whereas storage at room temperature led to a decline in efficacy over time, emphasizing the importance of low-temperature storage to preserve the structural integrity and functionality of nanocarrier systems.

The chitosan-coated ATR-loaded cubosomes were examined for stability over three months at temperatures of 4 and −8 °C. The withdrawn samples showed that the selected formulation was characterized by good stability with insignificant (*p* > 0.05) changes in the particle size, PDI, or zeta potential (Table 8). The stability of the formulation is attributed to the unique characteristics of these unilamellar vesicles and the metastability of their bulk cubic phase [77]. Poloxamer 407, present in the lamellar bilayer, forms a cap that coats the cubic bilayer, preventing the hydrocarbon chains from coming into contact with water and thus averting the aggregation and flocculation of cubosomes, supporting their colloidal stability [95]. Moreover, the chitosan coating provided adequate kinetic stability to the cubosomes. In a study conducted by Liu et al., the presence of chitosan and alginate coatings as stability-enhancing agents protected the nanoliposomes against heat, pH, and enzymatic damage more efficiently and preserved their intact structure compared to the uncoated nanoliposomes [96].

## 4. Conclusions

Nanoparticle-based drug delivery systems present a promising approach to conventional drug dosage forms due to their ability to enhance drug bioavailability and overcome permeability limitations. Chitosan is a biocompatible, biodegradable, and non-toxic polymer. Due to its physicochemical and biological properties, it has been widely used for developing and preparing nanocarriers. Furthermore, cubosomal formulations represent one of the promising liquid crystalline nanoparticles. Due to their significant hydrophobic region, cubosomes offer significant merits over other nanoparticles, such as a long shelf life, high stability, and high loading capacity for hydrophobic drugs, while also being capable of incorporating hydrophilic drugs. Cubosomes also offer the potential to deliver anticancer agents through targeted delivery and overcoming drug resistance. This study optimized chitosan-coated ATR-loaded cubosomes for colorectal cancer (CRC) treatment. The Box–Behnken design is a successful tool in optimizing the ATR-loaded cubosomes with smaller particle size and PDI, while maximizing the zeta potential. The chitosan-coated cubosomes demonstrated a pH-dependent release that was minimal at pH 1.2 and 4.5 and increased at pH 6.8 and 7.4, indicating the potential use of such a coated nanosystem for the targeted deliver of ATR to CRC. Atorvastatin-loaded cubosomes significantly affected HCT116 cell viability at concentrations of 1 and 5 µM, with complete inhibition of viable cell count at a concentration of 5 µM. The effects of the drug were prominent even in the presence of chitosan coating. Therefore, the fabricated optimized chitosan-coated ATR-loaded cubosomes may be promising nanocarriers to deliver the drug to the colon area by avoiding drug degradation in the stomach and bypassing the systemic circulation, resulting in enhanced localized anticancer activity. Further studies are required to evaluate the cellular uptake mechanisms and in vivo antitumor activity of the cubosomal formulations in the treatment of colorectal cancer.

## Figures and Tables

**Table 1:** Conditions and constraints in the Box–Behnken experimental design for the optimization of Atorvastatin-loaded liquid crystalline lipid nanoparticles.

Independent Variables	Coded Level			Actual Values		
P407 (mg) (A)	−1	0	+1	200	300	400
PVA * (% *w*/*w*) (B)	−1	0	+1	0	2.5	5
**Dependent Variables**	**Goal**					
Particle size (nm) (Y1)	Minimize					
PDI ** (Y2)	Minimize					
Zeta potential (mV) (Y3)	Optimize					

* Polyvinyl alcohol (PVA), ** polydispersity index (PDI).

**Table 2:** The scheme applied in the in vitro release study at different pH values to stimulate the gastrointestinal tract conditions [43].

Media	pH	Resembling Fluid	Incubation Time
Hydrochloric acid	1.2	SGF	0–2 h
Phosphate buffer	4.5	SGF + SIF	2–4 h
Phosphate buffer	7.4	SIF	4–6 h
Phosphate buffer	6.8	SCF	6–8 h

Simulated gastric fluid (SGF), simulated intestinal fluid (SIF), and simulated colonic fluid (SCF).

**Table 3:** Experimental matrix and observed responses from the randomized runs in the Box–Behnken design for atorvastatin-loaded cubosomes *.

	Factor 1	Factor 2	Response 1	Response 2	Response 3
Run	P407 Weight (mg)	PVA ** Conc. (% *w*/*w*)	Size (nm)	PDI ***	Zeta Potential (mV)
1	300	2.5	235.70 ± 11.35	0.280 ± 0.04	−20.90 ± 2.41
2	300	0	248.11 ± 1.25	0.228 ± 0.05	−24.60 ± 1.58
3	200	0	169.15 ± 4.14	0.310 ± 0.02	−22.98 ± 0.82
4	300	2.5	235.40 ± 8.55	0.301 ± 0.05	−21.50 ± 2.23
5	200	5	175.98 ± 1.24	0.352 ± 0.02	−18.40 ± 2.70
6	300	5	242.56 ± 1.25	0.282 ± 0.02	−18.60 ± 0.29
7	400	2.5	120.00 ± 1.66	0.136 ± 0.01	−22.20 ± 1.05
8	400	5	130.15 ± 5.77	0.147 ± 0.07	−19.70 ± 0.31
9	200	2.5	163.80 ± 1.25	0.320 ± 0.01	−22.20 ± 0.85
10	300	2.5	240.85 ± 10.12	0.279 ± 0.08	−20.40 ± 2.32
11	400	0	128.20 ± 0.01	0.149 ± 0.01	−23.80 ± 0.36
12	300	2.5	238.70 ± 9.58	0.290 ± 0.05	−21.20 ± 2.52

* Data are shown as mean ± SD (*n* = 3), ** polyvinyl alcohol (PVA), *** polydispersity index (PDI).

**Table 4:** Model terms evaluation of Box–Behnken design of atorvastatin-loaded cubosomes.

Factors	Variables	Standard Error	VIF *	Ri^2^ **
A	Poloxamer 407 (mg)	0.4082	1	0.000
B	Polyvinyl alcohol (% *w*/*w*)	0.4082	1	0.000

* Variance inflation factor (VIF), ** coefficient of determination (Ri^2^).

**Table 5:** Statistical parameters for various responses of the formulated atorvastatin-loaded liquid crystalline lipid nanoparticles.

Response *	Model F-Value	Lack of Fit Value	*p*-Value	Predicted R^2^	Adjusted R^2^	Adequate Precision	Best-Fit Model
Y1	632.13	1.69	<0.0001	0.9867	0.9965	59.0981	Quadratic
Y2	34.74	5.29	0.0002	0.7783	0.9388	16.6558	Quadratic
Y3	39.93	2.63	<0.0001	0.8209	0.8762	16.5300	Linear

* Particle size; Y1, polydispersity index; Y2, zeta potential; Y3.

**Table 6:** Response values for the optimized atorvastatin-loaded liquid crystalline lipid nanoparticles as generated by Box–Behnken design software.

Response	Predicted	Experimental
Particle size (nm)	120.577	120.00 ± 1.655
PDI *	0.1513	0.136 ± 0.008
Zeta potential (mV)	−21.5	−22.2 ± 1.05

* Polydispersity index (PDI).

**Table 7:** Comparison of drug-release kinetic models based on correlation coefficients (R^2^).

Kinetic Model	Equation	R^2^ Value	Best-Fit Model
Zero Order	Qt = Q0 + k0t	0.7569	No
First Order	logQt = logQ0 − k1t/2.303	0.6387	No
Hixson-Crowell	W01/3 − Wt1/3 = K	0.3890	No
Higuchi	Qt = kHt1/2	0.8059	No
Korsmeyer–Peppas	Mt/M∞ = kKtn	0.9546	Yes

**Table 8:** Physicochemical characteristics, namely particle size, polydispersity index (PDI), and zeta potential for the freshly prepared atorvastatin-loaded cubosomes and the stored samples at different times and temperature conditions *.

Time	Particle Size (nm)		PDI		Zeta Potential (mV)	
	4 °C	−8 °C	4 °C	−8 °C	4 °C	−8 °C
Day Zero	120.00 ± 1.66		0.136 ± 0.01		−22.2 ± 1.05	
First month	122.15 ± 2.12	129.45 ± 1.58	0.138 ± 0.74	0.159 ± 0.12	−20.8 ± 1.32	−21.45 ± 1.45
Second month	125.56 ± 1.35	133.14 ± 2.65	0.142 ± 0.55	0.178 ± 0.22	−20.05 ± 0.45	−21.18 ± 1.36
Third month	127.05 ± 1.23	135.85 ± 1.94	0.147 ± 0.33	0.183 ± 0.72	−21.62 ± 1.37	−22.9 ± 1.86

* Results are expressed as mean ± SD (*n* = 3).

## Data Availability

Data are available on request from the authors.

## Supplementary Materials

The following supporting information can be downloaded at https://www.mdpi.com/article/10.3390/pharmaceutics17060698/s1, Figure S1: Effects of chitosan-coated atorvastatin-loaded cubosome treatments on colorectal cancer cells. HCT116 were seeded in 96-well plates at a density of 5000 cells/well. Cells were treated with the indicated concentrations of (A) Atorvastatin, (B) cubosomes, (C) uncoated atorvastatin-loaded cubosomes, (D) chitosan-coated unloaded cubosomes, and (E) chitosan-coated atorvastatin-loaded cubosomes treatments. Results are expressed as a percentage of control and represent the average of three independent experiments ± SEM. Asterisk represents the concentration showing a significant decrease in cell density in treated versus control cells. Significance from the control is indicated by * *p* < 0.05 and ** *p* < 0.01, *** *p* < 0.001, and **** *p* < 0.0001. Figure S2: Effect of atorvastatin-loaded chitosan-coated cubosomes treatment on the viable and dead cell counts in colorectal cancer cells. HCT116 cells were seeded in 24-well plate at seeding density of 20,000 cell/well in triplicates. Cells were treated with the indicated concentrations of (A) Atorvastatin, (B) unloaded chitosan-coated cubosomes, (C) Atorvastatin-loaded chitosan-coated cubosomes, (D) cubosomes, and (E) Atorvastatin-loaded uncoated cubosomes treatments. Trypan blue dye exclusion assay results are expressed as a cell count and represent the average of three independent experiments ± SEM. The asterisk represents the concentrations showing a significant decrease in live cell count in treated versus control cells. Significance from the control is indicated by * *p* < 0.05 and ** *p* < 0.01, *** *p* < 0.001, and **** *p* < 0.0001.

## Abbreviations

The following abbreviations are used in this manuscript:

ATR	Atorvastatin
CRC	Colorectal cancer
PDI	Polydispersity index
P407	Poloxamer 407
PVA	Polyvinyl alcohol
GMO	glyceryl monooleate
FTIR	Fourier transform infrared spectroscopy
GIT	Gastrointestinal tract
SGF	Simulated gastric fluid
SIF	Simulated intestinal fluid
SCF	Simulated colonic fluid
IC50	Half-maximal inhibitory concentration
NPs	Nanoparticles
ANOVA	Analysis of variance
VIF	Variance inflation factor
SPSS	Statistical package for the social sciences
EE	Entrapment efficiency
R^2^	Coefficient of determination
SRB	Sulforhodamine B
PLGA	Poly (lactic-co-glycolic acid)
ELISA	Enzyme-linked immunosorbent assay
