# Supplementary material for: Development of Chitosan-Coated Atorvastatin-Loaded Liquid Crystalline Nanoparticles: Intersection of Drug Repurposing and Nanotechnology in Colorectal Cancer Management
Source: Pharmaceutics. 2025 May 27;17(6):698. doi: 10.3390/pharmaceutics17060698 (PMC12196457; doi:10.3390/pharmaceutics17060698)
Supplement: Supplementary file 1 [file pharmaceutics-17-00698-s001.zip › pharmaceutics-3594990-supplementary.pdf]

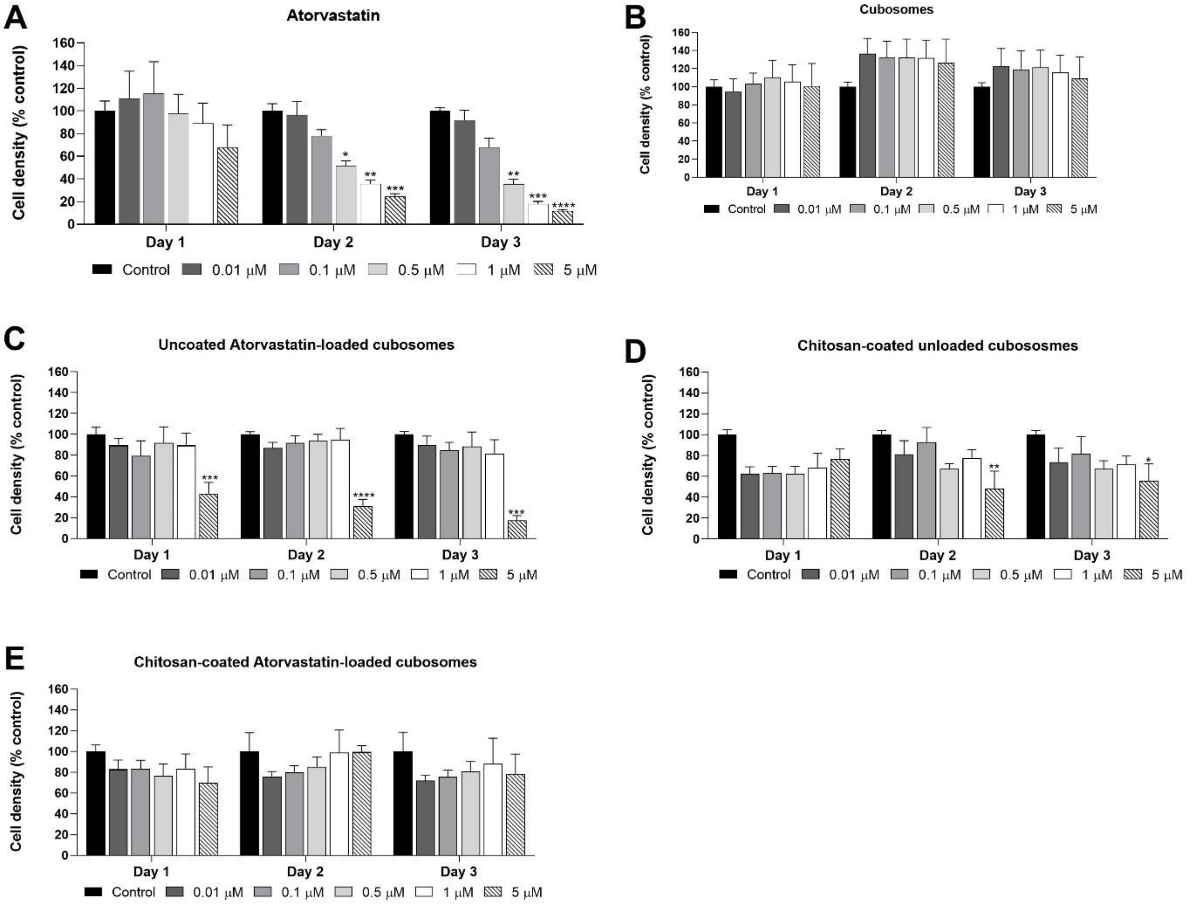

Figure S1: Effects of chitosan-coated atorvastatin-loaded cubosome treatments on colorectal cancer cells. HCT116 were seeded in 96-well plates at a density of 5,000 cells/well. Cells were treated with the indicated concentrations of (A) Atorvastatin, (B) cubosomes, (C) uncoated atorvastatin-loaded cubosomes, (D) chitosan-coated unloaded cubosomes, and (E) chitosan-coated atorvastatin-loaded cubosomes treatments. Results are expressed as a percentage of control and represent the average of three independent experiments ± SEM. Asterisk represents the concentration showing a significant decrease in cell density in treated versus control cells. Significance from the control is indicated by * $P < 0.05$ and ** $P < 0.01$, *** $P < 0.001$, and **** $P < 0.0001$.

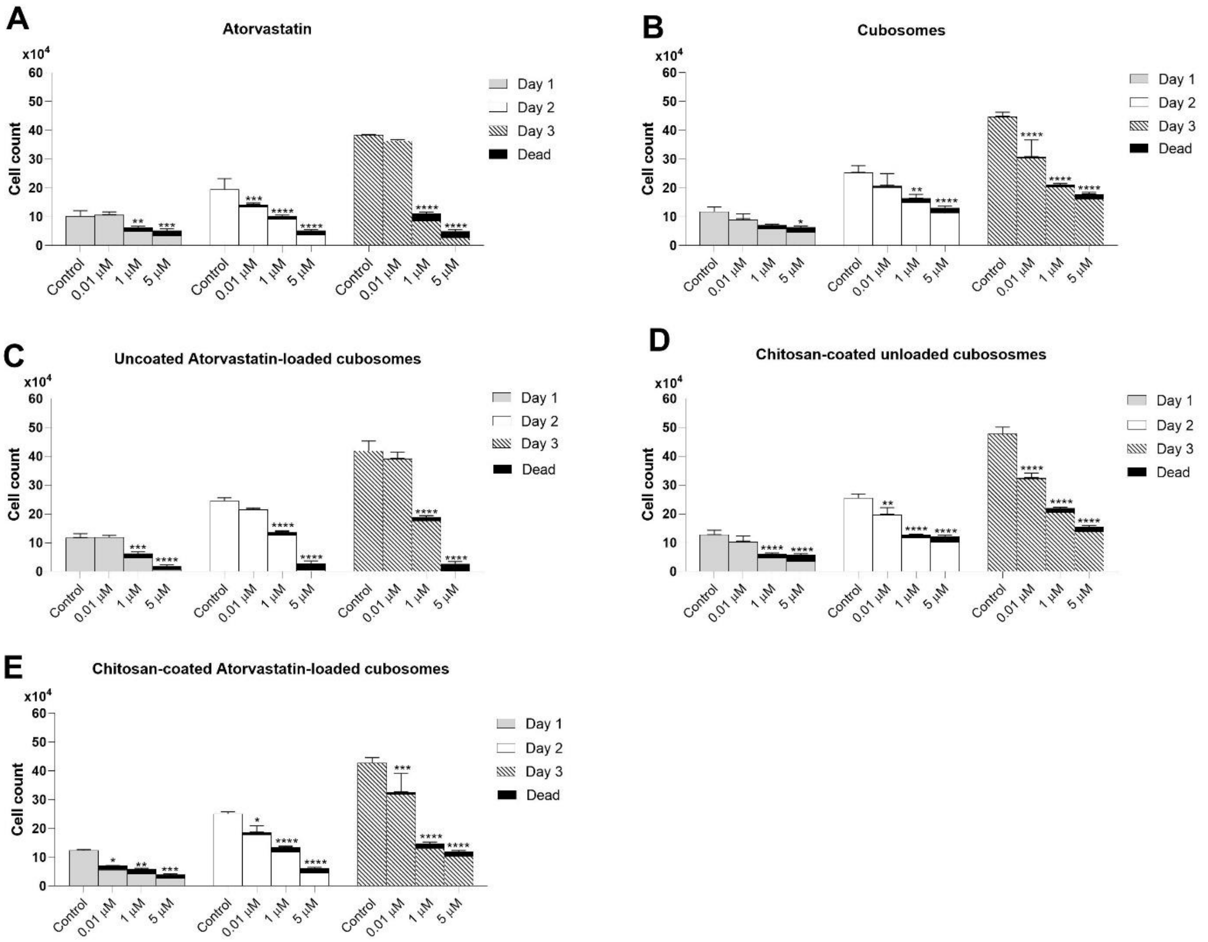

Figure S2: Effect of atorvastatin-loaded chitosan-coated cubosomes treatment on the viable and dead cell counts in colorectal cancer cells. HCT116 cells were seeded in 24-well plate at seeding density of 20,000 cell/well in triplicates. Cells were treated with the indicated concentrations of (A) Atorvastatin, (B) unloaded chitosan-coated cubosomes, (C) Atorvastatin-loaded chitosan-coated cubosomes, (D) cubosomes, and (E) Atorvastatin-loaded uncoated cubosomes treatments. Trypan blue dye exclusion assay results are expressed as a cell count and represent the average of three independent experiments ± SEM. The asterisk represents the concentrations showing a significant decrease in live cell count in treated versus control cells. Significance from the control is indicated by * $P < 0.05$ and ** $P < 0.01$, *** $P < 0.001$, and **** $P < 0.0001$.
